# Baseline CD44v6-positive circulating tumor cells to predict first-line treatment failure in patients with metastatic colorectal cancer

**DOI:** 10.18632/oncotarget.27794

**Published:** 2020-11-10

**Authors:** Chiara Nicolazzo, Flavia Loreni, Salvatore Caponnetto, Valentina Magri, Anna Rita Vestri, Rita Zamarchi, Angela Gradilone, Antonella Facchinetti, Elisabetta Rossi, Enrico Cortesi, Paola Gazzaniga

**Affiliations:** ^1^Department of Molecular Medicine, Liquid Biopsy Unit, Sapienza University of Rome, Rome, Italy; ^2^Department of Radiological, Oncological and Pathological Sciences, Sapienza University of Rome, Rome, Italy; ^3^Department of Public Health and Infectious Diseases, Sapienza University of Rome, Rome, Italy; ^4^Veneto Institute of Oncology IOV-IRCCS, Padua, Italy; ^5^Department of Surgery, Oncology and Gastroenterology, University of Padova, Italy

**Keywords:** cancer stem cells, CD44v6, circulating tumor cells, colorectal cancer, CellSearch^®^

## Abstract

CD44v6, the CD44 isoform mostly involved in cancer cell migration and invasion, has been identified as a functional biomarker and therapeutic target in colon cancer stem cells. We here provide evidence that baseline CD44v6-positive CTC predict treatment failure in patients with metastatic colorectal cancer undergoing first-line chemotherapy. We suggest that CD44v6-positive CTC can be used to early detect intrinsic drug resistance in this cancer type.

## INTRODUCTION

Cellular heterogeneity within circulating tumor cells (CTC) has been widely described and blood-monitoring studies in tumor patients have revealed the presence of CTC able to survive chemotherapy and to generate metastases after xenotransplantation in immunodeficient mice [1]. Therefore, the identification of drug resistant CTC in the entire pool of tumor cells disseminated in the bloodstream would, at least theoretically, provide a unifying hypothesis on CTC and cancer stem cells (CSC). Nevertheless, the relationship between circulating tumor cells and cancer stem cells is complex and currently under debate and the discovery of specific markers is an aim hard to be reached [2]. CD44v6, the CD44 isoform mostly involved in cancer cell migration and invasion, has been identified as a functional biomarker of stemness and therapeutic target in colorectal cancer (CRC) tissues [3]. CD44v6 expression has been reported in all colorectal cancer stem cells, and it is required for their migration and generation of metastatic tumors [4]. This concept is supported by experimental mouse models, which demonstrated that tumorigenic activity is confined in the CD44v6 population [3].

Functional studies have recently demonstrated that patient-derived colorectal CTC bear all the functional attributes of CSC and are strongly enriched for CD44v6 expression [5]. From a clinical perspective, CD44v6 is a negative prognostic factor in CRC, being 5-years survival rate of patients with CD44v6-positive and -negative tumors 52.78% and 80.95% respectively [6]. Furthermore, CD44v6 contributes to chemoresistance as demonstrated by *in vitro* studies showing that CD44v6-overexpressing cells are resistant to 5-fluorouracil (5-FU) or oxaliplatin by activating PI3K/Akt, mitogen-activated protein kinase/extracellular-signal-regulated kinase (MAPK/ERK), epithelial-mesenchymal transition (EMT), and autophagy-related signaling pathways [7–10]. Whether CD44v6 might be used to reflect the burden of circulating drug-resistant cells before starting therapy remains an unanswered question. We conducted a pilot study in order to evaluate the prognostic significance of baseline CD44v6-positive CTC in metastatic colorectal cancer patients candidate to first-line treatment.

## RESULTS

**Figure 1:**
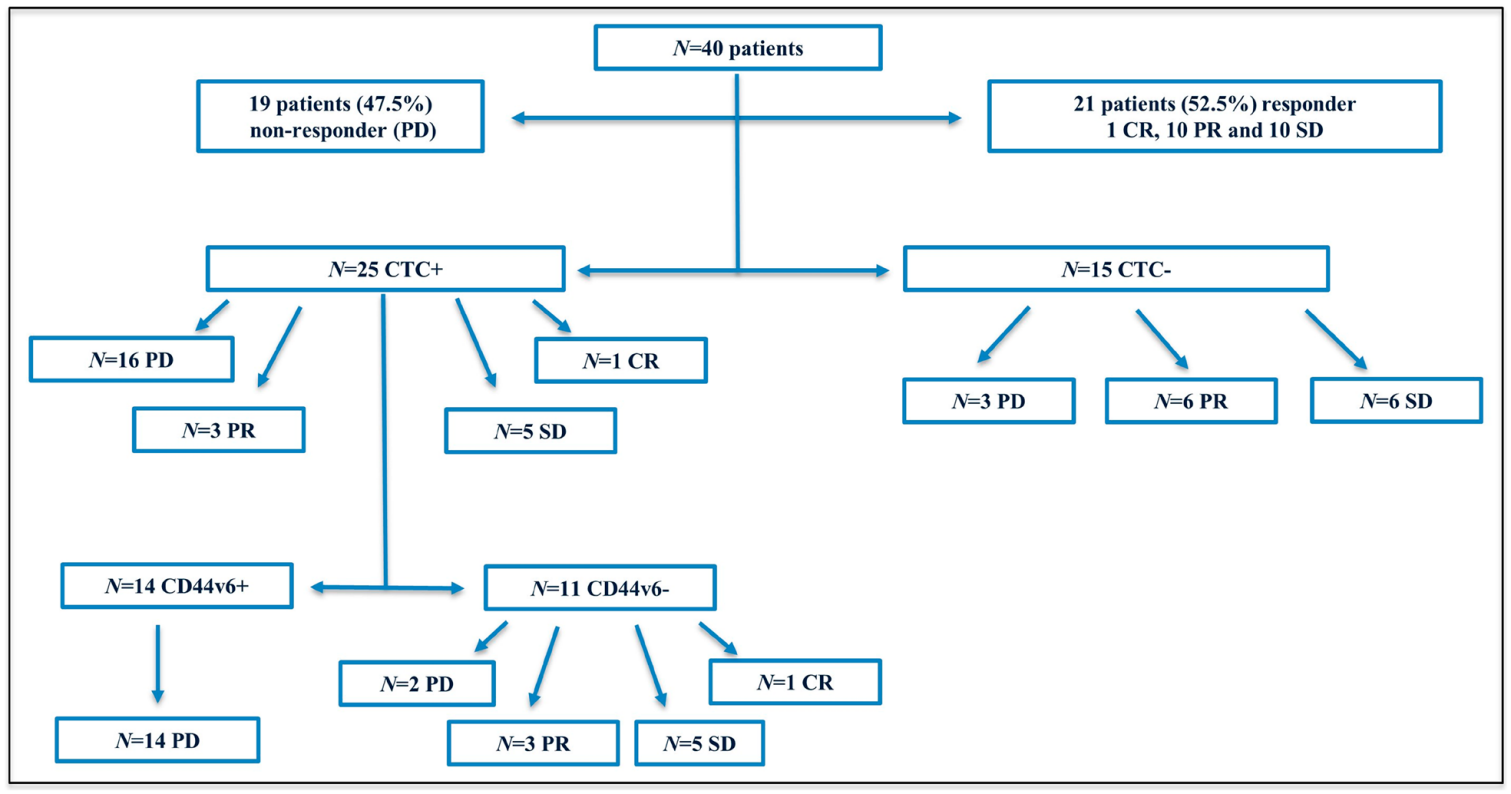
Flow diagram of the study. Abbreviations: N, number; PD, progressive disease; CR, complete response; PR, partial response; SD, stable disease; CTC, circulating tumor cells; +, positive; –, negative.

In order to assess the CD44v6 status in CTC from metastatic colorectal cancer (mCRC) patients, blood samples from 40 patients were analyzed through CellSearch^®^ system before starting first-line therapy. Baseline characteristics of enrolled patients are listed in Table 1. Tumor response was measured 12 months after start of treatment using the revised Response Evaluation Criteria in Solid Tumors (RECIST) v1.1. Patients with stable disease (SD), partial response (PR) or complete response (CR) were regarded as responders, whilst patients with progressive disease (PD) as non-responders. Tumor responses (SD, PR or CR) were observed in 21 out of 40 patients (52.5%) with 1 CR, 10 PR and 10 SD, whereas no response (PD) was observed in 19 patients (47.5%). The study flow diagram is shown in Figure 1. Based on the distribution of CTC, patients were dichotomized into two groups, as follows: negative (0 CTC) and positive (≥ 1 CTC). In regard to CD44v6 status, a threshold of ≥ 1 CTC expressing the antigen was applied to consider a sample as positive. Patients were 20 women and 20 men. No difference was found between mean age (female yrs 64.1 ± 7.7, median 65; men 62.8 ± 10.1, median 63). There was a statistical difference between CTC and sex (Fisher’s exact test *p* < 0.001). CTC-positive patients were 18 women (72%) and 7 men (28%), while CTC-negative were 2 women (13.3%) and 13 men (86.7%). No statistical difference was observed between CD44v6 expression and sex (4 men and 10 women in CD44v6-positive group vs 3 men and 8 women in CD44v6-negative group).

**Table 1:** Patient characteristics at baseline

Characteristics	No. of patients (*n* = 40)
**Sex**	
Male	20 (50%)
Female	20 (50%)
**Primary tumor location**	
Right	10 (25%)
Left	19 (47.5%)
Rectum	11 (27.5%)
**Stage of disease**	
Metastatic	40 (100%)
**Metastatic site**	
Liver	40 (100%)
**RAS/BRAF status (tumor tissue)**	
Wild type	17 (42.5%)
Mutant	16 (40%)
unknown	7 (17.5%)
**Line of therapy**	
1st	40 (100%)
**Therapy**	
FOLFOXIRI /bevacizumab	7 (17.5%)
FOLFIRI/bevacizumab	1 (2.5%)
FOLFOX/bevacizumab	8 (20%)
FOLFIRI/EGFRi	14 (35%)
FOLFOXIRI/EGFRi	3 (7.5%)
FOLFOX	3 (7.5%)
FOLFIRI	1 (2.5%)
XELOX	1 (2.5%)
XELIRI	2 (5%)
**CTC = 0**	15 (37.5%)
**CTC ≥ 1**	25 (62.5%)
**CD44v6-positive**	14 (35%)
**CD44v6-negative**	11 (27.5%)

### Circulating tumor cells and CD44v6 status

**Figure 2:**
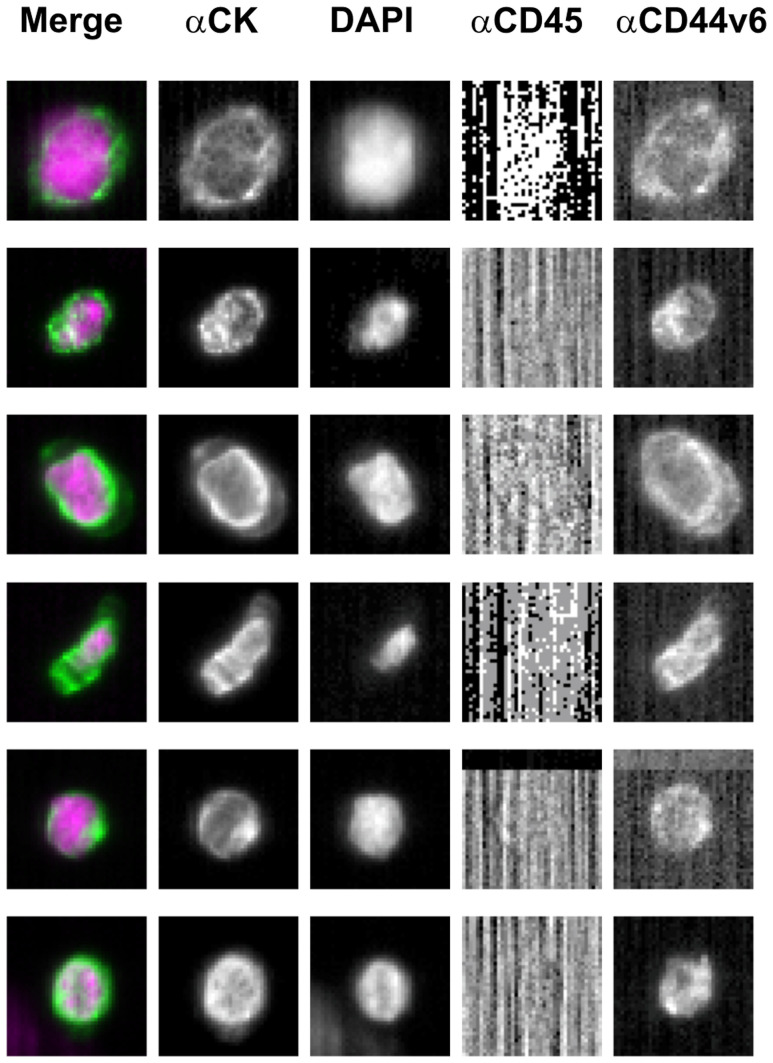
Representative images of CD44v6-positive circulating tumor cells isolated from one metastatic colorectal patient. Abbreviations: CK-FLU, cytokeratin fluorescein-conjugated (green); DAPI, 4′,6-diamidino-2-phenylindole (violet); APC, allophycocyanin; PE, phycoerythrin.

CTC were detected in 25 out of the 40 (62.5%) patients evaluated, whilst no CTC were found in 15 patients (37.5%). Among the 25 patients with CTC, 4 (16%) had 1 CTC, 5 (20%) had 2 CTC, 4 (16%) had 3 CTC, 3 (12%) had 4 CTC, 2 (8%) had 5 CTC, 1 (4%) had 6 CTC, 2 (8%) had 7 CTC, 2 (8%) had 8 CTC, 1 (4%) had 10 CTC and 1 (4%) had 11 CTC (range 1-11, median number 3). CD44v6 expression was found in 14 out of the 25 (56%) CTC-positive patients, whereas no CD44v6 expression was detected in 11 out of 25 (44%) patients. In the group of CD44v6-positive patients, CD44v6 expression was found in all CTC (100%) in 3 out of 14 patients, in 50% of CTC in 3 out of 14 and in more than half of CTC (≥ 50%) in 8 out of 14 (range 1–8, median number 4). Figure 2 shows 6 CD44v6-expressing CTC isolated from one patient. CTC numbers and related CD44v6 status for each patient are listed in Supplementary Table 1.

### Circulating tumor cells according to treatment regimen

Among the 33 patients for whom RAS status of the primary tumor was available, 16 patients received chemotherapy plus antiangiogenic drugs while 17 received chemotherapy plus epidermal growth factor receptor (EGFR) inhibitors (see Supplementary Table 1). CTC were found more frequently in patients with RAS mutant primary tumors compared to wild-type (94% versus 41% respectively). Accordingly, CD44v6-positive CTC were found in 73% of patients who received antiangiogenic drugs, compared to 14% of patients candidate to receive EGFR inhibitors. CTC were found in 3/7 (42%) patients who did not receive targeted therapies; 2/3 (67%) expressed CD44v6.

### Circulating tumor cells and clinical response

In the group of CTC-positive patients we had 16 out of 25 (64%) patients with progression of disease at 12 months, 5 out of 25 (20%) with stable disease, 3 out of 25 (12%) with partial response, while 1 out of 25 (4%) had a complete response. In the group of CTC-negative patients we had PD in 3 out of 15 (20%) patients, SD in 6 out of 15 (40%), PR in 6 out of 15 (40%) and no patients had CR (0%). We found a statistical difference between progressive disease and CTC (Fisher’s exact test *p* = 0.01; PHI index = 0.427 *p* = 0.007). The association between CTC and clinical response is shown in Table 2.

**Table 2:** Clinical response and CTC number or CD44v6 status at baseline

	Patients							
	CTC+		CTC−		CD44v6+		CD44v6−	
Response	N	%	N	%	N	%	N	%
**PD**	16	64	3	20	14	100	2	18
**PR**	3	12	6	40	0	0	3	27
**SD**	5	20	6	40	0	0	5	45
**CR**	1	4	0	0	0	0	1	9

Abbreviations: N, number; PD, progressive disease; PR, partial response; SD, stable disease; CR, complete response; CTC, circulating tumor cell; +, positive; −, negative.

### Relation of CD44v6 status in circulating tumor cells to clinical response

All patients who presented with at least 1 CTC expressing CD44v6 had progression of disease at 12 months (100%), whilst no SD, PR or CR were observed. On the other hand, in the group of CD44v6-negative patients, we had PD in 2 out of 11 (18%) patients, and SD in 5 out of 11 (45%), PR in 3 out of 11 (27%) and CR in 1 out of 11 (9%). The association between progressive disease and CD44v6 was found statistically significant (Fisher’s exact test *p* < 0.0001; PHI index = 0.846 *p* < 0.00001). In Table 2 the association between CD44v6 expression and clinical response is shown.

## DISCUSSION

Several studies have provided evidence that CTC represent a heterogeneous pool of tumor cells, and that a subpopulation showing an intermediate phenotype between epithelial and mesenchymal might express stem-like markers. Consistently CTC expressing putative biomarkers of stemness have been associated to therapy failure and disease progression [11]. CD44, a widely expressed adhesion molecule, contributes to cell–cell and cell–matrix adhesion, cell growth and differentiation, and is highly expressed on stem cells [3]. It exists in numerous variant isoforms among which CD44v6 has been recognized as a marker for colon cancer CSC. There is growing evidence that CD44v6 is associated with an increased metastatic risk, drug resistance and a lower survival rate in different cancer types, nevertheless evidence about the prognostic significance of CD44v6-positive CTC is lacking to date. This pilot study was aimed to assess the association between CD44v6 and response to first-line regimen in metastatic colorectal cancer patients, evaluated according to RECIST criteria. At this regard, a follow up period of 12 months was considered appropriate, since first-line treatment with both doublets, FOLFIRI or FOLFOX, produces a median progression-free survival (PFS) of 8.5-9 months in most randomized phase III trials [12, 13]. As far as we know this is the first demonstration that baseline CD44v6-positive CTC are associated to treatment failure, suggesting that CD44v6-positive subpopulations may reflect a biomarker of intrinsic resistance to treatment. Despite the small sample size, we observed that the characterization of CD44v6 in CTC at baseline is more significantly associated to treatment failure than CTC enumeration. In this respect, the choice to assess the prognostic significance of CTC irrespective of the standard cut-off (3 CTC/7.5 mL) was based on our previous demonstration that the presence of at least 1 CTC at baseline count is more predictive for poor prognosis in mCRC patients [14].

Whether CD44v6 expression in CTC might be attributed to the acquisition of stem-like properties is still an unanswered question. Recently, Fumagalli et al. demonstrated in a colorectal cancer model that CSC are not present in circulation, and that non-CSC are in fact the major seeding cells, while conversion to CSC at the metastatic site is required for efficient metastatic outgrowth [15].

Nevertheless, our results are in line with those described by other authors, who found CD44v6 expression in CTC cultured lines and demonstrated the existence of patient-derived colorectal CTC that bear all the functional attributes of CSC [16, 17]. Although these models are promising in drug-screening and resistance mechanism research, they are technically challenging, time-consuming and costly, thus not suitable for routine clinical practice. In addition, the rate of CTC in colon cancer patients is low, making not easy to establish CTC cultures in this tumor type.

The identification of CD44v6 in CTC might represent a useful tool for better evaluation of tumor response. Since CD44v6 acts as a co-receptor for vascular endothelial growth factor (VEGF) [18], the detection of CD44v6-positive CTC might be used to serially monitor the emergence of drug resistance in course of anti-angiogenic therapy. At this regard, we observed a higher frequency of CTC-positive patients in the group of RAS mutant compared to RAS wild-type, and CD44v6-positive CTC were also more frequent in patients candidate to receive antiangiogenic drugs compared to those who were treated with EGFR inhibitors. The treatment failure prediction of CD44v6-positive circulating tumor cells seemed independent from the first-line treatment regimen, although the sample size did not allow drawing significant conclusions. Furthermore, the activity of immune checkpoint inhibitors in microsatellite instability (MSI)-high CRC might be reduced in CD44v6-positive tumors, due to the interference of Fas signaling by CD44 variant isoforms [19]; consequently, a further application of CD44v6-positive CTC might be immunotherapy monitoring. In addition, the CD44v6 test in CTC might be used to identify high-risk stage II/III patients who could benefit from additional systemic therapies after primary tumor surgery.

This work has several limitations. First, further studies with a larger number of patients are required to understand the statistical significance of these findings as well as to clarify whether CD44v6 might really reflect the burden of CRC stem cells in this tumor type. Second, since treatment regimen was inconsistent among patients, a future study enrolling a larger patients’ population is planned to clarify whether CD44v6 might reflect an intrinsic resistance to most conventional chemotherapies/targeted therapies or to specific anticancer drugs. Third, despite the aim of this study was to correlate CD44v6-positive CTC and response to first-line regimen, a longer follow up time, which is in course, will allow us to correlate CD44v-positive CTC with PFS and overall survival (OS). Further studies are required to confirm whether this test might improve the early identification of metastatic colorectal cancer patients who are expected to poorly respond to first-line treatments.

In conclusion, CD44v6 seems to act as a drug resistance signature in CTC from metastatic colorectal cancer patients, being associated to poor tumor response to different first-line treatments. Despite the small number of patients, which is consistent with a pilot study, its expression in CTC at baseline seems to reflect a wide mechanism of drug intrinsic resistance, which might be suggestive for stem-like properties. We speculate that the detection of CD44v6-positive CTC might represent an additional prognostic tool useful to early identify patients with an expected poor response to first-line regimens.

## MATERIALS AND METHODS

### Experimental design

We conducted a pilot study in order to evaluate the utility of baseline (pre-treatment) assessment of CD44v6 status in CTC in predicting clinical outcomes in mCRC patients. The first step was developing a CD44v6-specific assay by integrating CellSearch^®^ system (Menarini Silicon Biosystems, Castel Maggiore, Bo, Italy) with a monoclonal antibody direct against the stemness marker. After that, a blood sample from each patient enrolled in the study was collected before starting the first-line treatment and used for analysis of CTC. Response to treatment was based on imaging documentation, mostly CT scans, and coded according to RECIST version 1.1.

### Patients and healthy volunteers

Forty patients with mCRC were enrolled at baseline before start of first-line treatment. The protocol had been approved by Ethical Committee of Policlinico Umberto I of Rome (protocol n. 668/09, July 09, 2009; amended protocol 179/16, March 01, 2016). Blood from healthy volunteers was taken for spiking experiments. Informed consent was obtained from all participants included in the study. Eligible patients had metastatic colorectal cancer surgically unresectable and/or metastatic (stage IV). Other inclusion criteria were: over 18 years of age; ECOG Performance Status ≤ 2; adequate bone marrow, liver and renal function. Systemic anticancer therapy was triplet (FOLFOXIRI) or doublet (FOLFOX or FOLFIRI) backbone chemotherapy with bevacizumab or EGFR inhibitors upon the decision made by the physician. Patients were monitored for 12 months.

### Circulating tumor cells analysis

A volume of 7.5 mL of peripheral blood from each participant was collected into CellSave Preservative tubes (Menarini Silicon Biosystems), kept at room temperature and processed through CellSearch^®^ system within 72 h. To this end, the CellSearch^®^ CXC kit (Menarini Silicon Biosystems) was employed and anti-human CD44v6 Phycoerythrin (PE)-conjugated antibody (clone FAB3660P; R&D Systems, Minneapolis, USA) was added into user-defined marker channel at a concentration of 0.02 μg/mL. Briefly, after epithelial cellular adhesion molecule (EpCAM)-based immunoselection, the enriched cells were labelled with fluorescent dyes for the detection of nucleus, cytokeratins (CK) 8, 18, and 19, CD44v6 and CD45. Immunofluorescence image were finally analyzed through CellSearch^®^ Analyzer II. An event was considered as a CTC when having round to oval morphology, a visible nucleus, positive staining for CK, negative staining for CD45, and negative or positive staining for CD44v6.

### Development and optimization of CTC assay specific for CD44v6 expression

**Figure 3:**
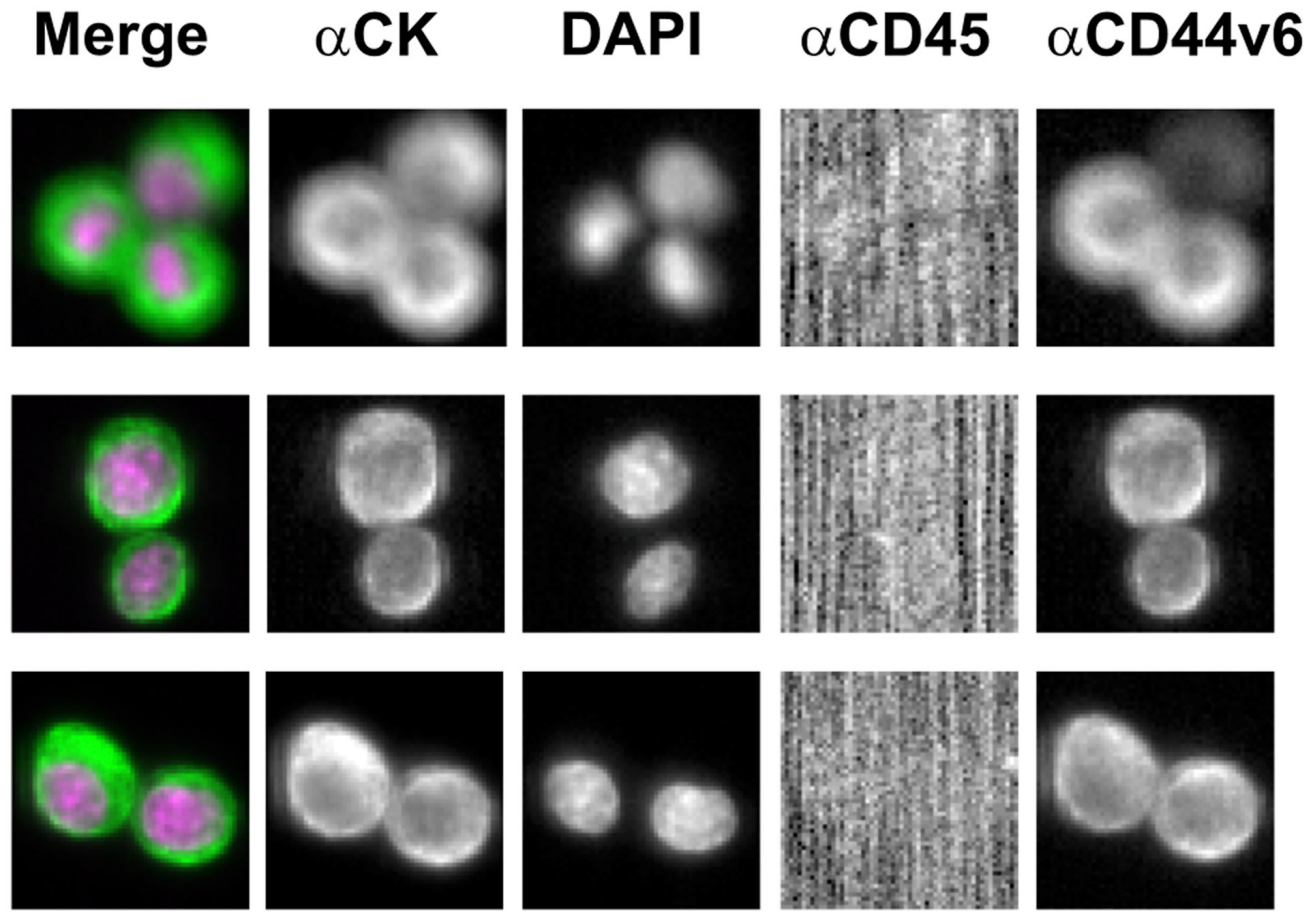
Illustrative images of CD44v6 immunostaining of MDA-MB-231 cell line through CellSearch^®^ system (antibody concentration: 0.02 μg/mL; Exposure Time: 0.4 sec). Abbreviations: CK-FLU, cytokeratin fluorescein-conjugated (green); DAPI, 4′,6-diamidino-2-phenylindole (violet); APC, allophycocyanin; PE, phycoerythrin.

In order to optimize the antibody concentration and the Exposure Time of the image scanning [20–24], spiking experiments in blood samples from healthy volunteers were performed using a cell line known to express CD44v6. To this purpose, MDA-MB 231 cell line exhibiting the antigen in close to 90% of the population at low fluorescence intensity, but with a good resolution of signal to background was employed. It was selected the minimum antibody concentration required to obtain a positive result. Briefly, the antibody stock solution (25 μg/mL) was first dissolved into CXC dilution buffer (Menarini Silicon Biosystems) to get a ratio of 1:75. Then, for one sample, 30 μL of diluted antibody solution was brought to a final volume of 450 μL by addition of the same buffer. Images of PE channel were acquired using an Exposure Time of 0.4 second. A clear image, with a dark background (black or nearly black), was considered as a positive result. Figure 3 shows a positive signal in MDA-MB231 cells without increasing non-specific background.

### Statistical analysis

A sample size of 40 was enrolled as cohort for assessment of CD44v6 status in CTC in mCRC patients undergoing first-line chemotherapy. Based on the presence (≥ 1) or absence of CTC, patients were dichotomized into two categories (positive vs negative). Similarly, in regard to CD44v6 status, a threshold of ≥ 1 CTC expressing the antigen was applied to consider a sample as positive. Continuous data were summarized by mean, median and standard deviation. Categorical data were reported by counts and percentages. To assess the difference of age we used Mann-Whitney test. The exact Fisher’s test was used to compare the categorical data; the phi index was used to highlight associations between markers and status of disease. The probability level was *p* < 0.05. Statistical analysis was performed using STATA v.12.

## SUPPLEMENTARY MATERIALS



## Abbreviations

5-FU: 5-fluorouracil
APC: allophycocyanin
CK: cytokeratins
CR: complete response
CSC: cancer stem cells
CTC: circulating tumor cells
DAPI: 4′,6-diamidino-2-phenylindole
EGFR: epidermal growth factor receptor
EMT: epithelial-mesenchymal transition
EpCAM: epithelial cellular adhesion molecule
FLU: fluorescein
MAPK/ERK: mitogen-activated protein kinase/extracellular-signal-regulated kinase
mCRC: metastatic colorectal cancer
MSI: microsatellite instability
OS: overall survival
PD: progressive disease
PE: Phycoerythrin
PFS: progression-free survival
PR: partial response
RECIST: Response Evaluation Criteria in Solid Tumors
SD: stable disease
VEGF: vascular endothelial growth factor
