# Supplementary material for: Baseline CD44v6-positive circulating tumor cells to predict first-line treatment failure in patients with metastatic colorectal cancer
Source: Oncotarget. 2020 Nov 10;11(45):4115–22. doi: 10.18632/oncotarget.27794 (PMC7665234; doi:10.18632/oncotarget.27794)
Supplement: Supplementary file 1 [file oncotarget-11-4115-s001.pdf]

Baseline CD44v6-positive circulating tumor cells to predict first-line treatment failure in patients with metastatic colorectal cancer

SUPPLEMENTARY MATERIALS

Supplementary Table 1: Treatment regimen, CTC counts and CD44v6 status in metastatic colorectal cancer patients

Patient[#]	CTC	CD44v6-positive CTC	Treatment regimen
1	5	4	Folfoxiri/bevacizumab
2	0	/	Folfiri/cetuximab
3	6	0	Folfiri/cetuximab
4	3	0	Folfiri/cetuximab
5	0	/	Folfiri/cetuximab
6	11	8	Folfoxiri/bevacizumab
7	0	/	Folfiri/cetuximab
8	2	0	folfox
9	8	4	Folfoxiri/bevacizumab
10	1	0	Folfiri/cetuximab
11	0	/	xeliri
12	2	0	Folfox/bevacizumab
13	4	2	Folfoxiri/bevacizumab
14	0	/	Folfiri/cetuximab
15	7	5	Folfoxiri/bevacizumab
16	3	2	Folfox/bevacizumab
17	0	/	Folfiri/panitumumab
18	4	0	Folfox/bevacizumab
19	0	/	Folfiri/cetuximab
20	0	/	Folfiri/cetuximab
21	1	0	Folfoxiri/cetuximab
22	1	0	Folfox/bevacizumab
23	0	/	folfox
24	0	/	Folfoxiri/cetuximab
25	2	2	folfiri
26	2	0	Folfoxiri/cetuximab
27	0	/	Folfiri/cetuximab
28	3	2	Folfox/bevacizumab
29	0	/	Folfiri/cetuximab
30	4	0	Folfoxiri/bevacizumab
31	8	6	xelox
32	0	/	Folfiri/bevacizumab

33	2	0	Folfiri/cetuximab
34	10	5	Folfox/bevacizumab
35	5	4	Folfoxiri/bevacizumab
36	0	/	folfox
37	7	4	Folfox/bevacizumab
38	0	/	xeliri
39	1	1	Folfiri/cetuximab
40	3	3	Folfox/bevacizumab
